# Supplementary material for: Sex differences in outcomes from mild traumatic brain injury eight years post-injury
Source: PLoS One. 2022 May 27;17(5):e0269101. doi: 10.1371/journal.pone.0269101 (PMC9140230; doi:10.1371/journal.pone.0269101)
Supplement: S1 Table — (DOCX) [file pone.0269101.s001.docx]

**S1 Table.** **Descriptive and inferential statistics for the males and females in the mTBI (8-years post-injury) and comparison groups for items on the outcome measures with a significant main effect or interaction of Sex RPQ, HADS Anxiety, PCL-C, WLQ Time demands, WLQ Output demands).**

|  | **mTBI at 8 years**  **M (SD)** | | **Comparison**  **M (SD)** | | **Two way ANOVA** |  |  |
| --- | --- | --- | --- | --- | --- | --- | --- |
|  | **Males**  **(n=77)** | **Females**  **(n=74)** | **Males**  **(n=74)** | **Females**  **(n=77)** | **Group (F, sig, partial eta square)** | **Sex (F, sig, partial eta square)** | **Group x Sex (F, sig, partial eta square)** |
| **Post-Concussion Symptoms (RPQ**) (df = 1, 298). (Bonferroni corrected p = .003) | | | | | |  |  |
| Headaches | 1.09 (1.27) | 1.59 (1.30) | 0.54 (1.00) | 1.06 (1.45) | **13.69, p<.001, ηp^2^=.04*** | **12.40, p<.001, ηp^2^=.04*** | 0.01, p=.94, ηp^2^<.01 |
| Dizziness | 0.61 (1.08) | 1.07 (1.26) | 0.23 (0.75) | 0.58 (1.08) | **12.53, p<.001, ηp^2^=.11*** | **11.07, p<.001, ηp^2^=.04*** | 0.18, p=.68, ηp^2^<.01 |
| Nausea | 0.40 (0.94) | 0.66 (1.10) | 0.14 (0.58) | 0.27 (0.77) | **10.78, p=.001, ηp^2^=.04*** | 3.94, p=.05, ηp^2^=.01 | 0.37, p=.54, ηp^2^<.01 |
| Noise sensitivity | 0.66 (1.18) | 1.16 (1.50) | 0.32 (0.80) | 0.69 (1.02) | **9.41, p=.002,** **ηp^2^=.03*** | **10.65, p=.001, ηp^2^=.03*** | 0.26, p=.61, ηp^2^<.01 |
| Sleep problem | 1.19 (1.38) | 1.73 (1.58 | 1.11 (1.27) | 1.43 (1.34) | 1.46, p=.23, ηp^2^<.01 | 7.09, p=.008, ηp^2^=02 | 0.45, p=.51, ηp^2^<.01 |
| Fatigue | 1.35 (1.44) | 1.88 (1.50) | 0.86 (1.25) | 1.44 (1.39) | 8.21, p=.004, ηp^2^=.03 | **11.76, p<.001, ηp^2^=.04*** | 0.02, p=.88, ηp^2^<.01 |
| Irritable | 1.00 (1.34) | 1.27 (1.26) | 0.59 (1.06) | 0.66 (1.05) | **13.82, p<.001, ηp^2^=.04*** | 1.54, p=.22, ηp^2^<.01 | 0.55, p=.46, ηp^2^<.01 |
| Depressed | 0.87 (1.15) | 1.20 (1.34) | 0.82 (1.26) | 0.65 (1.04) | 4.68, p=.03, ηp^2^=.02 | 0.32, p=.57, ηp^2^<.01 | 3.36, p=.07, ηp^2^=.01 |
| Frustrated | 1.10 (1.36) | 1.28 (1.30) | 0.55 (1.01) | 0.84 (1.10) | **12.78, p<.001,** **ηp^2^=.04*** | 2.88, p=.09, ηp^2^=.01 | 0.16, p=.69, ηp^2^<.01 |
| Forgetfulness | 1.44 (1.36) | 1.76 (1.52) | 0.53 (1.11) | 0.79 (1.52) | **40.54, p<.001,** **ηp^2^=.12*** | 3.87, p=.05, ηp^2^= .01 | 0.03, p=.87, ηp^2^<.01 |
| Concentration | 1.04, (1.27) | 1.34 (1.35) | 0.55 (1.06) | 0.64 (1.12) | **18.25, p<.001,** **ηp^2^=.06*** | 1.88, p=.17, ηp^2^<.01 | 0.61, p=.44, ηp^2^<.01 |
| Thinking | 1.05 (1.23) | 1.43 (1.40) | 0.50 (1.01) | 0.53 (0.95) | **29.22, p<.001, ηp^2^=.09*** | 2.36, p=.13, ηp^2^<.01 | 1.68, p=.20, ηp^2^<.01 |
| Blurred vision | 0.48 (0.97) | 1.23 (1.41) | 0.32 (0.92) | 0.34 (0.81) | **18.85, p<.001, ηp^2^=.06*** | **9.98, p=.002, ηp^2^=.03*** | **9.29, p=.003, ηp^2^=.03*** |
| Light sensitivity | 0.64 (1.17) | 1.16 (1.44) | 0.35 (0.94) | 0.52 (1.07) | **11.86, p<.011,** **ηp^2^=.04*** | 6.64, p=.01, ηp^2^=.02 | 1.76, p=.19, ηp^2^<.01 |
| Double vision | 0.21 (0.68) | 0.59 (1.20) | 0.11 (0.56) | 0.14 (0.56) | **9.14, p=.003,** **ηp^2^=.03*** | 5.34, p=.02, ηp^2^=.02 | 3.73, p=.06, ηp^2^=.01 |
| Restlessness | 0.86 (1.23) | 1.18 (1.33) | 0.43 (0.94) | 0.56 (0.97) | **16.09, p<.001, ηp^2^=.05*** | 2.93, p=.09, ηp^2^=.01 | 0.55, p=.46, ηp^2^<.01 |
| **HADS Anxiety** (df 1,298) (Bonferroni corrected p=.007) | | | | | | | |
| Tense | 0.75 (0.73) | 0.80 (0.70) | 0.77 (0.56) | 0.86 (0.70) | 0.24, p=.62, ηp^2^<.01 | 0.71, p=.40, ηp^2^<.01 | 0.08, p=.78, ηp^2^<.01 |
| Frightened | 0.53 (.074) | 0.89 (0.90) | 0.72 (0.87) | 0.89 (0.90) | 0.39, p=.53, ηp^2^<.01 | 5.80, p=.02, ηp^2^=.02 | 1.54, p=.22, ηp^2^<.01 |
| Worrying thoughts | 0.78 (0.83) | 0.97 (0.94) | 1.00 (0.91) | 1.04 (0.85) | 1.84, p=.18, ηp^2^<.01 | 1.49, p=.22, ηp^2^<.01 | 0.50, p=.48, ηp^2^<.01 |
| Relaxed | 0.77 (0.58) | 0.96 (0.84) | 0.62 (0.68) | 0,70 (0.71) | 6.15, p=.01, ηp^2^=.02 | 2.83, p=.09, ηp^2^<.01 | 0.49, p=.49, ηp^2^<.01 |
| Butterflies | 0.40 (0.63) | 0.59 (0.70) | 0.50 (0.60) | 0,64 (0.61) | 0.91, p=.34, ηp^2^<.01 | 5.03, p=.03, ηp^2^=.02 | 0.14, p=.70, ηp^2^<.01 |
| Restless | 0.92 (0.87) | 0.96 (0.90) | 0,73 (0.69) | 0.66 (0.77) | 6.87, p=.009, ηp^2^=.02 | 0.03, p=.87, ηp^2^<.01 | 0.32, p=.58, ηp^2^<.01 |
| Panic | 0.39 (0.73) | 0.82 (0.87) | 0.51 (0.75) | 0.70 (0.71) | 0.01, p=.99, ηp^2^<.01 | **12.54, p<.001, ηp^2^=.04*** | 1.97, p=.16, ηp^2^<.01 |
| **Post-traumatic stress disorder checklist: Civilian (PCL)** (df =1,298) (Bonferroni corrected p =.008) | | | | | | | |
| Disturbing thoughts | 1.47 (0.80) | 2.00 (1.30) | 1.64 (0.92) | 1.64 (0.81) | 0.76, p=.38, ηp^2^<.01 | 5.64, p=.02, ηp^2^=.02 | 5.59, p=.02, ηp^2^=.02 |
| Upset reminder | 1.52 (0.81) | 2.08 (1.21) | 1.73 (1.06) | 1.65 (0.81) | 0.96, p=.33, ηp^2^<.01 | 4.51, p=.04, ηp^2^=.02 | 8.02, p=.01, ηp^2^=.03 |
| Avoid activities | 1.43 (0.85) | 1.74 (1.20) | 1.43 (0.83) | 1.32 (0.55) | 4.17, p=.04, ηp^2^=.01 | 1.04, p=.31, ηp^2^<.01 | 4.32, p=.04, ηp^2^=.01 |
| Distant | 1.78 (1.02) | 1.97 (1.17) | 1.74 (1.09) | 1.75 (0.86) | 1.14, p=.29, ηp^2^<.01 | 0.73, p=.40, ηp^2^<.01 | 0.59, p=.44, ηp^2^<.01 |
| Irritable | 1.61 (0.85) | 1.91 (1.02) | 1.58 (0.94) | 1.56 (0.72) | 3.41, p=.07, ηp^2^=.01 | 1.79, p=.18, ηp^2^<.01 | 2.43, p=.12, ηp^2^<.01 |
| Concentrate | 1.86 (1.00) | 2.08 (1.20) | 1.51 (0.88) | 1.68 (0.91) | **10.51, p=.001, ηp^2^=.03** | 2.79, p=.10, ηp^2^<.01 | 0.07, p=.79, ηp^2^<.01 |
| **Work limitations: Time demands (**df = 1, 171) (Bonferroni corrected p=.01) | | | | | | | |
| Hours | 6.87 (11.27)^a^ | 18.91 (24.58)^b^ | 11.67 (17.35) ^c^ | 11.49 (21.72) ^d^ | 0.21, p=.65, ηp^2^<.01 | 4.19, p=.04, ηp^2^=.03 | 4.44, p=.04**,** ηp^2^=.03 |
| Get going | 10.29 (18.15) | 19.08 (24.96) | 13.89 (21.02) | 13.16 (19.05) | 0.13, p=.72, ηp^2^<.01 | 1.59, p=.21, ηp^2^<.01 | 2.22, p=.14, ηp^2^=.01 |
| Start | 5.5 (11.61) | 9.87 (21.39) | 10.00 (19.51 | 7.89 (21.04) | 0.20, p=.66, ηp^2^<.01 | 0.16, p=.69, ηp^2^<.01 | 1.30, p=.26, ηp^2^<.01 |
| Not stop | 11.50 (19.70) | 25.00 (33.39) | 17.78 (20.38) | 18.57 (25.25) | 0.01, p=.98, ηp^2^<.01 | 3.44, p=.07, ηp^2^=.02 | 2.72, p=.10, ηp^2^=.02 |
| Routine | 10.50 (16.04) | 17.95 (26.87) | 8.33 (15.29) | 9.21 (19.64) | 3.21, p=.08, ηp^2^=.02 | 1.87, p=.17, ηp^2^=.01 | 1.16, p=.28, ηp^2^<.01 |
| **Work limitations: Output Demands** (df = 1,171) (Bonferroni corrected p=.01) | | | | | | | |
| Workload | 6.73 (13.21) | 14.10 (21.30) | 10.10 (14.42 | 10.26 (19.63) | 0.08, p=.93, ηp^2^<.01 | 2.12, p=.15, ηp^2^=.01 | 1.95, p=.16, ηp^2^=.01 |
| Fast | 6.37 (13.08) | 16.44 (21.96) | 8.15 (14.00) | 7.89 (16.55) | 1.81, p=.18, ηp^2^=.01 | 8.82, p=.05, ηp^2^=.02 | 4.23, p=.04, ηp^2^=.02 |
| Finish | 5.50 (11.61) | 11.49 (24.00) | 8.51 (15.00) | 9.61 (17.11) | 0.05, p=.83, ηp^2^<.01 | 1.73, p=.19**,** ηp^2^=.01 | 0.82, p=.37, ηp^2^<.01 |
| No mistakes | 5.77 (10.64) | 11.25 (20.37) | 5.77 (10.64) | 11.25 (20.37) | 0.46, p=.46, ηp^2^<.01 | 1.31, p=.25**,** ηp^2^<.01 | 1.18, p=.28, ηp^2^<.01 |
| Capable | 7.21 (15.13) | 18.75 (27.00) | 10.11 (19.27) | 13.46 (24.90) | 0.14, p=.71, ηp^2^<.01 | 5.25, p=.02, ηp^2^=.02 | 1.58, p=.21, ηp^2^<.01 |

^a^n=51, ^b^n=37, ^c^n=45, ^d^n=37 as only participants in employment completed this measure.
