# Supplementary material for: Sex differences in outcomes from mild traumatic brain injury eight years post-injury
Source: PLoS One. 2022 May 27;17(5):e0269101. doi: 10.1371/journal.pone.0269101 (PMC9140230; doi:10.1371/journal.pone.0269101)
Supplement: Attachment — Submitted filename: Response to Reviewers.docx [file pone.0269101.s008.docx]

Response to the reviewer

1. There are still minor orthographic mistakes, such as spaces, double points, etc. The authors should thoroughly double check this or it should be done by PLOS One during the final editing.

We have checked this again and corrected all of the issues we could find including the reference format and punctuation around the brackets.

2. Within the introduction (page 4, line 67) the term mild TBI should be replaced by mTBI.

We have corrected this

3. The sentence (page 5, line 73) should start with a capital letter, thus MTBI.

Corrected

4. NZ population (page 5, line 80) should be introduced as New Zealand… when used for the first time.

Corrected

5. Content-wise: the intro (p.4-5, lines 67-72) should be clarified as the following sentence is somehow confusing: “Most probably the authors meant that the initial 38.1% of mixed anxiety and/or depressive symptoms decreased by 6% between year 1 and 4 after mTBI. Why did the authors mention the community samples, is this sample a mixed sample of TBI positive and negative humans? It would be particularly of interest whether the portion of psychiatric symptoms was increased in comparison to the general population. Please, could the authors clarify this point?

Apologies for this – we agree it doesn’t make sense and I suspect it happened when we shortened the introduction. Your interpretation is correct, rates decreased by 6% between 1- and 4-years post-injury. It was not a mixed TBI and community sample. At 4 years post-injury the rates of anxiety and depression were similar to those observed in community samples.

We have now reworded it as follows and added details about rates for anxiety, depression, and comorbid illness separately (pages 4-5 lines 71- 76):

“38.1% of the sample met the cut-offs for anxiety and or depression on the HADS at 12 months post-injury (2.2% depression, 25.5% anxiety, 10.2% depression and anxiety) which decreased to 32.1% four years post-injury (7.7% depression only, 13.6 % anxiety only and 11.8% depression and anxiety), similar to rates observed in community samples.”

6. Methods (page 8, lines 141-143): as mTBI was not clearly defined according to the ACRM criteria, this should be stated in the limitation part (lack of information on the time of loss of consciousness). I am very sorry for not having this mentioned before. Please see references: Medicine ACoR. Definition of mild traumatic brain injury. Journal of Head Trauma Rehabilitation. 1993;8(3):86-87; Silverberg ND, Iverson GL. Expert Panel Survey to Update the American Congress of Rehabilitation Medicine Definition of Mild Traumatic Brain Injury. Archives of physical medicine and rehabilitation. 2021;102(1):76-86;

Thank you for the citations. We have added the following to the limitations section (p 36, line 598-600):

“contact. In addition, we used WHO criteria to define mTBI, not the American Congress of Rehabilitation, which includes the presence of observable signs and subjective symptoms (dizziness, confusion) [60, 61]. “

Please let me know if you need any additional information,

Yours sincerely,

Professor Nicola Starkey

Email: nstarkey@waikato.ac.nz
