# Supplementary material for: Sex differences in outcomes from mild traumatic brain injury eight years post-injury
Source: PLoS One. 2022 May 27;17(5):e0269101. doi: 10.1371/journal.pone.0269101 (PMC9140230; doi:10.1371/journal.pone.0269101)
Supplement: Attachment — Submitted filename: Reviewer comments to author 02 10 2021.pdf [file pone.0269101.s003.pdf]

Reviewer Comments to the authors:

Dear authors,
thank you very much for your scientific effort to elucidate long-term outcome after mild TBI (mTBI), which is an underestimated neuropsychiatric burden, and thus this work will help closing the gap of evidence in the field.

Please see my comments regarding weaknesses and my suggestions that might help to improve clarity of your manuscript.

- The study design needs to be clarified, as it seems to be a mixed design including retrospective data from a previous study (RPQ and HADS from 1- and 12-months after mTBI) and a cross-sectional analysis at 8-years after mTBI. Maybe I missed something, but in the current form it remains somehow unclear or even misleading. It is well known that long-term follow up after TBI is challenging due to immense loss of data/follow up, thus this idea of combining data in principle is good enough but should be clearly stated. As there were only early results at 1- and 12-months post-mTBI available for the RPQ and HADS this should be the primary outcome over time, and thus the study design should be clarified throughout the title, abstract and whole MS as mentioned above.
- It remains unclear why sample sizes are different (mTBI: N=151, controls: N=211), and it is important to have a control group which does not differ in terms of educational level and ethnicity, both relevant factors in terms of neuropsychiatric burdens after TBI.
- The definition of mTBI should be more precise according to the ACMR criteria with negative CCT scans. If these ACMR criteria were not used, it should be better explained and clarified. Alternatively, definitions which were used in the CENTER-TBI (uncomplicated, complicated mTBI) or TRACK-TBI study could be used and information on CCT/cMRI would be desirable.
- Mild TBI should be used as mTBI instead MTBI
- There is a need of explanation on the rationale for including adolescents aged > 16 years instead of merely adults. Were the 16-year-olds from the school or working population at the time of their TBI? Sometimes inclusion was > 16, sometimes ≥ 16 years; this should be adopted. However, I recommend including adults ≥ 18 years of age at TBI.
- Inclusion criteria should be specified, as it seems that also repetitive mTBI patients were included, these patients might hamper results and should be excluded,

particularly as the pathophysiology of repetitive TBI and single TBI seems to differ according to the literature.

- The Methods part in terms of outcome measures should be more precise and I suggest the following organization:

  1. Neuropsychiatric burden measured by patient-reported outcome measures (PROMs: RPQ, HADS, PCL-C) instead of using “health”
  2. community participation (Participation Assessments with Recombined Tools)
  3. work (Work Limitations Questionnaire)

- I suggest using the common wording of PROMs
- All outcome measures should be revised and particularly cut-off scores should be given.
- The paragraphs on assessing *community participation* and *work* would benefit from further clarity. Maybe I missed something, but it remained unclear whether those parameters were assessed by self-rating or external ratings? Were the applied instruments validated for the assessed age groups? Further information would be helpful.
- I suggest to be careful with the diagnosis of PTSD as you only assessed PTSD symptoms with the PCL-C checklist.
- The abstract would benefit from more concise description of the study design, measurement time points, patient- or external-rated assessments, and clear statements for the three outcome measures
  1. Neuropsychiatric sequels measured by PROMs (RPQ, HADS, PCL-C)
  2. community participation (Participation Assessments with Recombined Tools)
  3. work (Work Limitations Questionnaire)
- The introduction would benefit from a better focus. Overall, the intro should be shorter with focus on mTBI and outcome with respect to neuropsychiatric burdens regarding RPQ, HADS, PTSD as well as community participation and work as well as sex differences; in the current stage the intro is broad and the reader gets somehow lost within lots of information which is not clearly focus on the presented study.
- The methods part is mixed with results (referral to Table 1). The same is relevant for Table 2, which is a result as well. I suggest to merely describe the methods which you used to get the controls. All details about the controls belong to the results part. I think it is a problem that controls and mTBI subjects differed in terms of education and ethnicity as stated above and this should be adopted when possible.
- Table 1 is a result and should be changed in terms of the column order, thereby it would be easier to anticipate which sample was included and which differed significantly (p-values should directly be incorporated). As the analyzed sample is not representative for the initial sample in terms of age, sex distribution and ethics, this is

not ideal for data interpretation, and thus results in the current format need to be handled with care and should merely be descriptive. Due to this major limitation, I suggest to get another statical support and recalculate the data by using imputation analysis, or ITT or completer analysis, which might strengthen the conclusions.

- Could you please improve resolution of images?
- Could you please provide an abbreviation list, e.g. for SES p. 5, line 87
- What is the rationale for defined age groups 16-44 and 45 +? This should be clarified.
- Which confounders were assessed to minimize gender factors that might influence the detrimental females' outcome such as financial inequity, social position, marital status, etc. between sexes?
- Some wording should be double checked, e.g. the "8-year anniversary", p. 13, line 239
- The stats part needs revision in terms of primary and secondary outcome measures as indicated above. It seems that there were also patients with more than a single mTBI, these should be analyzed separately and not included in this study. Was data normal distributed, descriptive analysis should be given.
- In the result part absolute numbers e.g. for the RPQ should be interpreted in terms of the given cut-offs in the literature. For example, it should be stated whether patients had a manifest post-concussion syndrome. For definitions see e.g. Riemann et al. 2021 (DOI: 10.3171/2020.9.PEDS20421).
- The current literature should be updated, e.g. include references from doi.org/10.1016/S1474-4422(17)30371-X; doi.org/10.1186/s12955-020-01391-3; doi.org/10.1007/s11357-020-00273-2
- I assume that the authors are English native speakers. However, some parts of the manuscript would benefit from scientific/ language editing aiming a concise scientific English language.

Thank you very much for your immense research effort.
