# Supplementary material for: Sex differences in outcomes from mild traumatic brain injury eight years post-injury
Source: PLoS One. 2022 May 27;17(5):e0269101. doi: 10.1371/journal.pone.0269101 (PMC9140230; doi:10.1371/journal.pone.0269101)
Supplement: Attachment — Submitted filename: Reviewer comments to editor 10 02 2022.pdf [file pone.0269101.s004.pdf]

Reviewer Comments to the editor:

Dear Dr. Sirén,

thank you very much for giving me the opportunity to review the revised version of the manuscript, now entitled **Sex differences in outcomes from mild traumatic brain injury eight years post-injury.**

The authors did an immense effort to improve this manuscript However, I have minor suggestions that would help to improve the manuscript further:

- Need of another orthographic check
- Another English native proofread in terms of some wording and grammar would be beneficial
- I suggest adapting the last part of the abstract and the conclusion by being as specific and congruent in terms of the key results and messages.
- I suggest differing between single mTBI and repetitive mTBI throughout the manuscript

Thanks for your patience,

Katrin Rauen

Dear authors,

thank you very much for your immense work and effort that have improved your manuscript significantly. However, I have minor suggestions that would help to improve the manuscript further:

- Please provide another orthographic check
- Please double check some wording and grammar by another scientific English native proofread
- I suggest adapting the last part of the abstract and the conclusion by being as specific and congruent in terms of the key results and messages.

- I suggest differing between single mTBI and repetitive mTBI throughout the manuscript

Thank you very much for your effort.
