# Supplementary material for: Sex differences in outcomes from mild traumatic brain injury eight years post-injury
Source: PLoS One. 2022 May 27;17(5):e0269101. doi: 10.1371/journal.pone.0269101 (PMC9140230; doi:10.1371/journal.pone.0269101)
Supplement: Attachment — Submitted filename: Reviewer comments to authors 10 02 2022.pdf [file pone.0269101.s005.pdf]

Reviewer Comments to the authors:

Dear authors,

thank you very much for your immense work and effort that have improved your manuscript significantly. However, I have minor suggestions that would help to improve the manuscript further:

- Please provide another orthographic check
- Please double check some wording and grammar by another scientific English native proofread
- I suggest adapting the last part of the abstract and the conclusion by being as specific and congruent in terms of the key results and messages.
- I suggest differing between single mTBI and repetitive mTBI throughout the manuscript

Thank you very much for your effort.
