# Supplementary material for: Sex differences in outcomes from mild traumatic brain injury eight years post-injury
Source: PLoS One. 2022 May 27;17(5):e0269101. doi: 10.1371/journal.pone.0269101 (PMC9140230; doi:10.1371/journal.pone.0269101)
Supplement: Attachment — Submitted filename: Reviewer comments to editor 16 04 2022.pdf [file pone.0269101.s006.pdf]

Reviewer Comments to the editor:

Dear Dr. Sirén, dear Anna-Lena,

thank you very much for giving me the opportunity to re-review the 2nd revised version of the manuscript, entitled **Sex differences in outcomes from mild traumatic brain injury eight years post-injury.**

The authors succeeded improving their manuscript significantly. However, I have still minor suggestions that would help to improve the manuscript further:

- There are still minor orthographic mistakes, such as spaces, double points, etc. The authors should thoroughly double check this or it should be done by PLOS One during the final editing. Please see further minor points that need corrections: Within the introduction (page 4, line 67) the term mild TBI should be replaced by mTBI. The sentence (page 5, line 73) should start with a capital letter, thus MTBI. NZ population (page 5, line 80) should be introduced as New Zealand… when used for the first time.
- Content-wise: the intro (p.4-5, lines 67-72) should be clarified as the following sentence is somehow confusing: "38.1% of the sample met the cut-offs for anxiety and/or depression on the HADS at 12 months post-injury (similar to rates observed in community samples in our previous research; 7.7% for depression, 13.6% for anxiety which decreased to 32.1% four 72 years post-injury"
  Most probably the authors meant that the initial 38.1% of mixed anxiety and/or depressive symptoms decreased by 6% between year 1 and 4 after mTBI. Why did the authors mention the community samples, is this sample a mixed sample of TBI positive and negative humans? It would be particularly of interest whether the portion of psychiatric symptoms was increased in comparison to the general population. Please, could the authors clarify this point?
- Methods (page 8, lines 141-143): as mTBI was not clearly defined according to the ACRM criteria, this should be stated in the limitation part (lack of information on the time of loss of consciousness). I am very sorry for not having this mentioned before. Please see references: Medicine ACoR. Definition of mild traumatic brain injury. Journal of Head Trauma Rehabilitation. 1993;8(3):86-87; Silverberg ND, Iverson GL. Expert Panel Survey to Update the American Congress of Rehabilitation Medicine Definition of Mild Traumatic Brain Injury. Archives of physical medicine and rehabilitation. 2021;102(1):76-86;

Besides of the definition of mild TBI, my comments are minor issues. Thus, I suggest that the authors can change those without another round of revision. I hope my comments are useful for your decision making.

Best,

Katrin

Dear authors,

thank you very much for your efforts that clarified your manuscript. However, I have still minor suggestions that would help to improve the manuscript further:

- There are still minor orthographic mistakes, such as spaces, double points, etc. The authors should thoroughly double check this or it should be done by PLOS One during the final editing. Please see further minor points that need corrections: Within the introduction (page 4, line 67) the term mild TBI should be replaced by mTBI. The sentence (page 5, line 73) should start with a capital letter, thus MTBI. NZ population (page 5, line 80) should be introduced as New Zealand… when used for the first time.
- Content-wise: the intro (p.4-5, lines 67-72) should be clarified as the following sentence is somehow confusing: "38.1% of the sample met the cut-offs for anxiety and/or depression on the HADS at 12 months post-injury (similar to rates observed in community samples in our previous research; 7.7% for depression, 13.6% for anxiety which decreased to 32.1% four 72 years post-injury"
  Most probably the authors meant that the initial 38.1% of mixed anxiety and/or depressive symptoms decreased by 6% between year 1 and 4 after mTBI. Why did the authors mention the community samples, is this sample a mixed sample of TBI positive and negative humans? It would be particularly of interest whether the portion of psychiatric symptoms was increased in comparison to the general population. Please, could the authors clarify this point?
- Methods (page 8, lines 141-143): as mTBI was not clearly defined according to the ACRM criteria, this should be stated in the limitation part (lack of information on the time of loss of consciousness). I am very sorry for not having this mentioned before. Please see references: Medicine ACoR. Definition of mild traumatic brain injury. Journal of Head Trauma Rehabilitation. 1993;8(3):86-87; Silverberg ND, Iverson GL. Expert Panel Survey to Update the American Congress of Rehabilitation Medicine

Definition of Mild Traumatic Brain Injury. Archives of physical medicine and rehabilitation. 2021;102(1):76-86;

Besides of the definition of mild TBI, my comments are minor issues. Thanks for your effort researching long-term outcome after mTBI that provides important information for patients, clinicians, and future research.
